# Navigating laboratory services quality in challenging environments: A perspective for implementation in small, low-income countries and post-conflict settings

**DOI:** 10.4102/ajlm.v2i1.48

**Published:** 2013-08-27

**Authors:** Edward M. Kamau

**Affiliations:** 1 Special Programme for Research and Training in Tropical Diseases, World Health Organization

## Abstract

The need to establish and maintain good laboratory practices is recognised universally. However, due to differences in resources available for health services in different countries, allocation of financial and human resources in poor countries is severely constrained. The constraints faced by poor countries call for innovative approaches that would guarantee the minimum acceptable quality while striving to meet the highest standards. In resource-limited setting, it may be justifiable to develop and use ‘fit for purpose’ quality standards based on internationally-recognised laboratory quality management frameworks or protocols.

## Introduction

### Background

Under ideal conditions, medical practitioners rely on the use of quality laboratory data for evidence-based medical decision-making. Public health programme officials also rely upon laboratory data in order to detect outbreaks of disease through laboratory surveillance, determine policy for the implementation of disease-control measures, monitor disease, and determine the impact of control programmes. Furthermore, there is a heightened awareness of the importance of public health and clinical laboratories in ensuring that society is protected from re-emerging infectious agents and recurrent epidemics and pandemics.^1^

Although the existence of laboratory infrastructure is a prerequisite for the generation of specific laboratory parameters, quality outcomes result from adherence to national laws and guidelines; and of course, appropriate leadership and management are critical. In addition to laws and regulations, the medical device industry, governments, World Health Organization, non-governmental organisations and professional societies should work together to develop quality standards leading to an improvement of testing and assurance with regard to the quality of laboratory data.^2^ These laws and systems help ensure that all laboratory testing is of the highest quality.

## Rationale for investing in constrained settings

The key driving force to strengthen laboratory capacity in resource-constrained countries is to increase accuracy and reliability of data for diagnosis, treatment and control of diseases. In most, if not all, of the small, low-income countries in Africa,^3^ there is an urgent and overwhelming need to address not only laboratory quality standards but also the inadequate technical and human capacity to deliver services, weak integration amongst disease control and prevention programmes and low appreciation of contemporary developments in laboratory diagnosis. Furthermore, some countries face an additional challenge with respect to their official and/or national language, and greater support for Francophone and Lusophone countries by providing training and documentation in the major languages of the continent will help to address inequities in health research, as well as creating a unique area of work for the African Society for Laboratory Medicine (ASLM). Supporting African nations should achieve the objectives of equal treatment and comparison of countries in similar situations.^4^

Low-income countries and post-conflict settings present the greatest challenges as well as opportunities in laboratory medicine. With the appropriate tools and implementation of standards, laboratory professionals can demonstrate the contribution that high quality laboratory services make in improving the health status of the population. The evidence for improvement would come from data on the detection, management, prevention and research for neglected diseases. In the area of neglected tropical disease, a modest investment is likely to have maximum impact. There may be no ‘one size fits all’ solution to challenges in laboratory service quality, and each country or situation needs thorough scrutiny, as routine approaches that have worked well elsewhere may not be appropriate. At the same time, major challenges remain: overcoming the main infectious diseases; inadequate research capacity; the increasing burden of non-communicable diseases; and a constantly changing economic situation. Countries or regions emerging from armed conflict are in dire need of quality laboratory services. There is also a need to motivate countries to prioritise laboratory services in their national health plans, to take advantage of new funding mechanisms and to commit more domestic resources to health care services.

The implementation process is based on the following quality systems approach:

1. Acknowledgement of the need to improve the laboratory services at all decision-making levels within countries and to articulate potential health and economic benefits.
2. Assessment of capacity, infrastructure and training needs.
3. National consensus meeting of all stakeholders, including the development of guidelines and policy documents.
4. Identification and designation of a national quality assurance laboratory or office and leadership structure.
5. Allocation of resources to the maintenance of quality requirements.
6. Development and provision of technical training and supervision.
7. Participation in national and regional external quality control programmes that promote the implementation of corrective and preventative action plans that can be validated.
8. Participation in an accreditation programme.

This step-wise process is not specific to addressing laboratory services in the target settings only; it would be applicable wherever there are efforts to improve the quality of laboratory testing for any disease.

## The quality systems approach

The implementation of quality practices requires a systematic approach in a comprehensive and coordinated effort to meet quality objectives. Quality assurance (QA) is focused on providing confidence that quality requirements will be fulfilled. In most resource-constrained countries, there has been little coordination towards objectives and almost no resources are available to implement activities that would ensure quality laboratory services.

**FIGURE 1:**
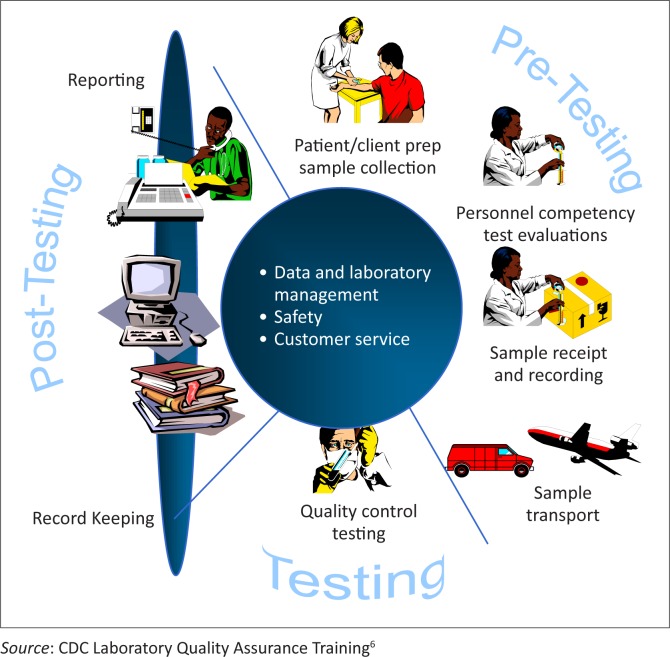
Model quality assurance cycle.

To achieve quality results, it is crucial that everyone who is involved in the process of laboratory testing is part of the quality process, from the person collecting specimens to the person making use of the test results. The purpose of a quality system is to avoid errors, provide consistent performance, ensure the integrity of data, increase efficiency and cost-effectiveness, provide customer satisfaction, training opportunities, and build credibility for the laboratory service on offer. In addition, all aspects of the testing process, covering the entire quality assurance cycle of pre-analysis, analysis and post-analysis (see Figure 1), must be addressed.

### Initiating a national laboratory quality system

The basis for addressing laboratory quality issues should be to develop a specific team with responsibility for implementing activities that will strengthen the capacity of the laboratory infrastructure. Based on information gathered from questionnaires or country-specific national laboratory services reports submitted by disease control units, and at the invitation of the Ministries of Health (MoH), stakeholders in laboratory services could initiate an assessment of laboratories by holding a meeting to review the country’s laboratory diagnosis action plan (if any is available) or developing such a plan for quality system implementation.^5^ This first step in implementing support for a quality laboratory system ensures a commitment on the part of the MoH toward strengthening laboratory capacity. This commitment is essential for effective and relevant change and to begin the process of addressing organisational structures that will contribute to the laboratory’s ability to provide quality services.

Subsequent to obtaining the appropriate commitment, a detailed assessment of the current laboratory system and practices in the country, which should give special attention to identifying the weakest link(s) in the quality system, will determine the most critical gaps and enable priorities to be established for addressing those gaps. The assessment should not only take into account the national, provincial and peripheral laboratory infrastructure, but should also determine the management and communication practices between the laboratory personnel and medical practitioners. Furthermore, relationships with the formal higher education sector (universities and medical training colleges) will need to be established, in order to influence and adopt curricula to meet the country’s needs and to engage these institutions in refresher and on-the-job training programmes. The assessment report should indicate clearly what actions are necessary and the time frame required for implementation of the quality system. More often than not, the first tangible activity would be to provide training on quality systems development to a core team of potential trainers. This is based on the realisation that most laboratory managers and technologists endeavour to provide quality results but have limited information regarding what steps need to be taken in order or to what benchmarks activities are to be performed.

In parallel with the above activities, it would be necessary to address other specific technical training needs such as the selection and validation of tests, training of those likely to perform laboratory testing and preparation for the establishment of a national integrated laboratory service. A major requirement would be to ensure that there are competent personnel who have the mandate to implement quality systems. Another critical component would be the creation or promotion of a central national laboratory to be the reference centre for the country or region with the responsibility of developing and reviewing standardised operating procedures (SOPs), selecting laboratory equipment and providing QA support, supervision, monitoring and evaluation.

## Basic characteristics in challenging environments

The following are essential features for the organisation and management of a quality laboratory system.

### Organisation

The organisation component of quality systems implementation involves the planning and organising of the quality programme, defining the scope of authority and responsibility of staff and the allocation of resources to sustain quality requirements. In addition, job descriptions, training and orientation, continuing professional education, and competence and performance appraisal would provide confidence that quality standards will be met and upheld.

### Equipment

Quality control (QC) of equipment includes selection of appropriate instruments and ensuring correct operation, providing for installation and initial calibration, and establishing maintenance mechanisms, including service contracts. Routine servicing, repairing and provision of information for troubleshooting and regular review of documentation are essential with regard to maximising the useful life of laboratory equipment.

### Purchasing and inventory

It is essential to define criteria for products and services to be purchased, as well as to establish a system for the receiving, inspecting, accepting or rejecting, storing and recording of all incoming and outgoing materials. The ability to assess and maintain the inventory, in addition to establishing a system to link supplies to their users, activities or specific records, would enhance the development of an efficient procurement management system.

### Records and process management

Establishing records and process management requires developing standardised document formats, systematic revision, approval and distribution, as well as managing patient test records, storage, retrieval and destruction systems. Providing an oversight on all laboratory operations such as methodology evaluation, validation procedures, SOPs, QC and external quality assessment are essential for process control.

### Information and occurrence (non-conformance) management

Information and occurenace management requires the management of incoming and outgoing information, standardisation of information capture, maintaining the confidentiality of patient information and ensuring competency in the appropriate information technology skills. Timely and effective resolution of laboratory errors minimises the negative effects that such occurrences can have on the integrity of laboratory services.

### Assessment, process improvement and customer focus

Regular evaluation of the entire QA cycle and the systematic evaluation of all laboratory procedures must be performed in order to ensure continued improvement in the quality of laboratory services. It also involves proactive gathering of information on customer satisfaction through surveys and feedback channels, as well as the use of said information to improve, recognise and reward the staff who provide a quality service.

### Personnel and work environment

The provision of adequate facilities, working and storage areas enhances reliable testing and ensures a safe working environment.

## Challenges

One of the main constraints associated with laboratory quality systems in challenging circumstances is the failure to realise that, as a public good, laboratory services should be achieved equitably and to the highest attainable level, making the case for the hard-to-reach populations a daunting task with regard to the provision of patient services that are comparable in quality to those offered elsewhere. Furthermore, there is often the reluctance to hold a routine laboratory to the same or higher standards as the reference laboratories, coupled with the common QA weaknesses that are found even in well-resourced settings. It is worth noting that a laboratory service system is only as strong as its weakest link and identifying the link(s) where there is potential for maximum impact of limited resources is not always straightforward.

## Conclusion

The strengthening or establishment of a laboratory services quality system in challenging environments and, in particular, in small, low-income countries or regions and those emerging from armed conflicts, is clearly an important goal and the activities outlined above are achievable. This approach is in line with the promotion of the point-of-care platform by ASLM for low income and lower-to-middle income countries, as it will require taking a step further up the ladder of hard-to-reach populations. For some of these settings, a modest improvement can help to address some of the complaints that may have precipitated the conflict whilst restoring devastated services and achieving the objective of equal treatment and comparison with countries or areas in similar situations. However, the implementation and improvements in laboratory services cannot be addressed in isolation. The use of evidence-based decision making in both clinical practice and in public health will require a change in attitude to one that values laboratory data. Finally, an improvement in laboratory infrastructure alone will not be beneficial unless similar or greater attention is given to the broader healthcare system.
